# Lipoprotein Receptors and Lipid Enzymes in Hepatitis C Virus Entry and Early Steps of Infection

**DOI:** 10.6064/2012/709853

**Published:** 2012-12-23

**Authors:** Eve-Isabelle Pécheur

**Affiliations:** ^1^Department of Mechanisms of Chronic Hepatitis B and C, Centre de Recherche en Cancérologie de Lyon, 69008 Lyon, France; ^2^Inserm U1052/CNRS UMR 5286, CRCL, Université de Lyon, 151 Cours Albert Thomas, 69424 Lyon Cedex 03, France

## Abstract

Viruses are obligate intracellular agents that depend on host cells for successful propagation, hijacking cellular machineries to their own profit. The molecular interplay between host factors and invading viruses is a continuous coevolutionary process that determines viral host range and pathogenesis. The hepatitis C virus (HCV) is a strictly human pathogen, causing chronic liver injuries accompanied by lipid disorders. Upon infection, in addition to protein-protein and protein-RNA interactions usual for such a positive-strand RNA virus, HCV relies on protein-lipid interactions at multiple steps of its life cycle to establish persistent infection, making use of hepatic lipid pathways. This paper focuses on lipoproteins in HCV entry and on receptors and enzymes involved in lipid metabolism that HCV exploits to enter hepatocytes.

## 1. Introduction

Hepatitis C is a chronic liver pathology affecting 170 million people worldwide, and 3 to 4 millions are newly infected each year. After a generally asymptomatic initial contamination, signs of liver injury appear within 20 to 30 years and lead to death from cirrhosis or hepatocellular carcinoma (HCC) in severe cases. At this stage the only therapeutic option is liver transplantation. 

A main source of contamination has been blood transfusion which peaked in the early 90s, until the hepatitis C virus (HCV) was discovered [1]. Epidemiological previsions report a steep increase of the HCV-related HCC incidence rate in western countries, till 2020–2025. The situation is worrying in emerging countries of Southeastern Asia, in China and Brazil with seroprevalence around 3 to 5%, and Central Africa and Egypt remain regions of very high endemicity, with a 25% prevalence in the latter. Hepatitis C is therefore a global health problem, with striking inequalities in the access to healthcare and implementation of treatments between world regions. In the absence of preventive vaccine, the actual standard of care treatment relies on a combination of interferon-alpha and ribavirin, to which direct-acting antivirals (DAA) can possibly be added. DAA molecules target viral enzymes of the replication complex and raise great hopes of viral eradication in the near future for treated patients. However, they induce viral resistance and severe adverse effects, and their costs are out of reach for patients of emerging countries. Treatments can be differently envisioned when considering hepatitis C not only as a liver pathology but also as a metabolic disease. Indeed HCV-infected patients very often display perturbations in glucose homeostasis, leading to insulin impaired signalling and resistance, likely to translate into accelerated liver disease progression and HCC occurrence. Dyslipidemias and hepatic steatosis are also clinically observed in association with HCV infection [2]. Interestingly, HCV entry into hepatocytes requires a subset of cell surface receptors and cofactors that, for some of them, are involved in lipoprotein metabolism or cholesterol transport. Recent studies have shown that lipid metabolic pathways are required for the entry, replication, and secretion of HCV [3]. The subversion at its own profit of lipid receptors by HCV at the entry step could profoundly and durably alter the lipid metabolic profile of infected cells. Therefore, therapies aimed at restoring normal lipid metabolism by targeting these receptors could be very useful at combating HCV infection.

In this paper we will focus on the role of lipoproteins, and of receptors and enzymes involved in lipid metabolism in HCV entry and infection. We will examine the peculiar composition of HCV particles, analyze how this relates to lipid receptor recognition at the hepatocyte cell surface, and finally delineate the role played by key enzymes of lipid metabolism in HCV infection. 

### 1.1. The Hepatitis C Virion: A Peculiar Arrangement of Lipids, Apolipoproteins, and Viral Proteins

**Figure 1:**
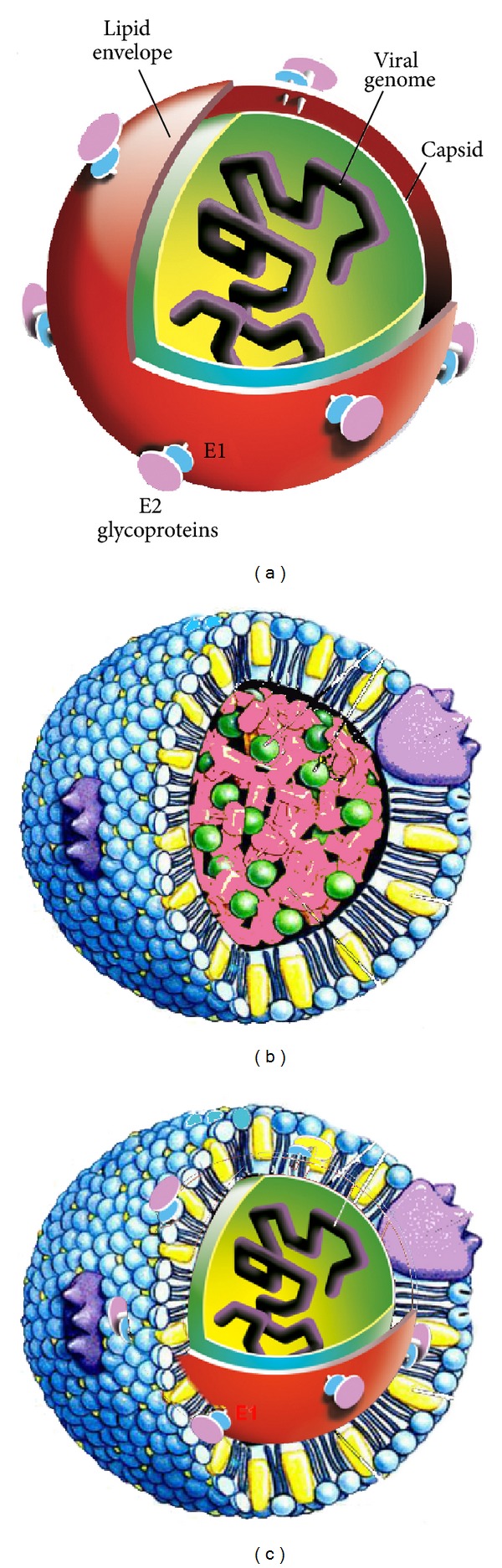
Schematic representations of the hepatitis C virus. (a) In the absence of three-dimensional structures of the virion or any of its structural proteins (the envelope proteins E1-E2 and the core protein), HCV is represented roughly as a sphere without any symmetry. The E1-E2 glycoproteins (blue and pink, resp.) are set in the viral envelope (red) *via* their transmembrane domains. Their distribution at the particle surface is left loose on purpose, since currently available high resolution cryo-TEM (transmission electron microscopy) examinations did not show any high-density layer of proteins on the lipid envelope [23, 24]. The internal layer is formed by the core protein (green) compacting the viral RNA genome (black); no structural information is available about the nucleocapsid and its arrangement (illustration by J.-F. Michel). *“This research was originally published in [8]*.” (b) Structural organization of a lipoprotein. The lipid core is composed of cholesterol esters (pink) and triglycerides (green); it is surrounded by a monolayer of phospholipids (blue), into which free cholesterol molecules are inserted (yellow). Apolipoproteins (purple) are inserted into the lipid layer of this particle. (c) Schematic representation of a lipo-viro-particle (LVP), where HCV nucleocapsid is embedded in the lipid core of the lipoprotein, harboring apolipoproteins and HCV E1-E2 glycoproteins at their surface.

Although the first cases of “non A non B” hepatitis were reported in the early 80s in polytransfused patients, and related to a viral antigen [4], the isolation and identification of the hepatitis C virus occurred only in 1989 [1]. HCV is a virus encoding a single-stranded RNA genome of positive polarity, and isolates are classified into 7 genotypes differing in nucleotide sequence by 30% to 35% [5, 6]. The viral particle is formed by the core protein compacting the viral RNA, surrounded by a lipid envelope harboring the glycoproteins E1 and E2 involved in viral entry and fusion (schematized in Figure 1(a)) (for specific reviews, see, for example, [5, 7, 8]). HCV belongs to the Flaviviridae family, which also comprises mosquito-borne viruses such as the emerging pathogens West Nile and Dengue flaviviruses, tick-borne encephalitis viruses, the cattle pestiviruses, and the newly classified pegiviruses [9]. Viruses of this family have a common genome organization, where the RNA serves as a template for the production of a large polyprotein posttranslationally processed into the individual structural proteins that build new virus particles, and nonstructural proteins that form the viral replication complex. Replication takes place in a network of membranes emanating from the endoplasmic reticulum (ER), the so-called membranous web, and viral particles are formed in the ER lumen. The replication machinery of HCV is composed of the non-structural proteins p7 (a viroporin), the NS2-3 protease, the NS3 serine protease and RNA helicase, the NS4A helicase, the NS4B and NS5A proteins, and the NS5B RNA-dependent RNA polymerase (RdRp) [5, 10]. Recent data established that p7 and NS2 act in concert to regulate virus assembly [11, 12]. 

Though related to other viruses of the Flaviviridae family, HCV greatly diverges from those on several aspects of its lifecycle, and some of its features are even unique. 

First, the only reservoirs for HCV are humans and chimpanzees. Second, the only cells reported to date for fully productive infection are human hepatocytes, although very recent data reported that endothelial cells of the blood-brain barrier supported HCV entry and replication [13]. Third, pioneering studies by Thomssen and coworkers revealed that HCV RNA isolated from sera of HCV-infected patients cofractionated with betalipoproteins in low to very low density fractions and could be coprecipitated by anti-beta-lipoprotein antibodies [14, 15]. This suggested a physical association between viral particles and elements of these lipoproteins (see also [16–18]). Ten years later, the group of Patrice André isolated low density particles from HCV-positive human sera and revealed the dual nature of these particles: viral and lipoprotein [19–21] (schematized in Figure 1). 

To understand the following, we will then quickly glance at the structure and composition of lipoproteins.

#### 1.1.1. Lipoprotein Composition

 Lipoproteins are a combination of lipids and proteins serving as lipid transporters in the blood. The lipid core is formed by cholesterol esters and triglycerides (Figure 1(b)), surrounded by a monolayer composed of free cholesterol and phospholipids. This particle is covered with proteins called apolipoproteins, exerting a dual role: contribution to lipoprotein structure (structuring role) and receptor recognition (signalling role) [22]. Human lipoproteins are classified into 4 groups, depending on their density: high, low, and very low density lipoproteins (HDL, LDL, VLDL, and chylomicrons, resp.). Each group has a different lipid and protein composition, defining its structure and function. Extensive details on this composition and on lipoprotein trafficking are beyond the scope of this paper; we will merely analyse the protein composition of each group, since the protein part of lipoproteins is involved in receptor recognition and subsequent signalling. HDLs are composed of apolipoproteins (apo) AI (main component), II, CI, II and III, D, and the exchangeable apoE. The main integral protein component of LDL is apoB100 (synthesized in the liver and termed apoB in the following), with one molecule of apoB *per* LDL particle. VLDLs harbor apoB, apoCI, II and III, and apoE at their surface, while chylomicrons are formed by apoAI and IV, apoB48 (of intestinal origin), apoCI, II and III, and apoE. ApoAI and apoB structure the lipoproteins which bear them, while others exchange between various lipoproteins (apoAII, C, and E).

**Figure 2:**
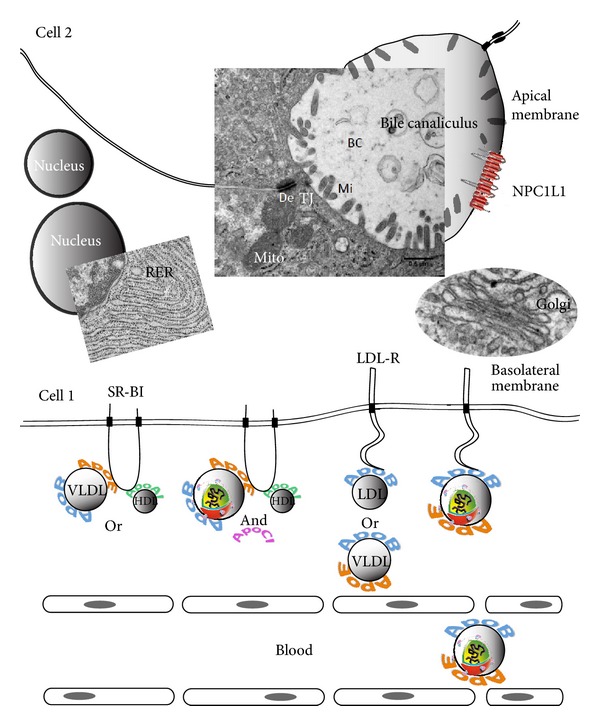
Architecture of human hepatocytes and proposed model for HCV LVPs. Interaction at the surface of the hepatocyte with their entry factors involved in lipid metabolism. For reasons of clarity, only lipoprotein receptors of HCV are indicated; also HCV is the only lipid particle depicted, transiting in the blood, where it would flow with lipoproteins. Two primary human hepatocytes are depicted (cells nos. 1 and 2), organized laterally around the apical membrane (bile pole). De, desmosome; TJ, tight junction; Mi, microvilli; Mito, mitochondria; RER, rough endoplasmic reticulum. Two nuclei per cell are represented, since hepatocytes are often bi-nucleated. HCV (depicted as an LVP) extravasates from blood to encounter the hepatocyte basolateral membrane. At this stage, it interacts with: (i) SR-BI in an apoE-dependent manner; this could be facilitated by HDL binding to their own attachement domain on SR-BI (through apoAI), and is most likely apoCI-dependent; (ii) LDL-R in an apoB-dependent manner. Normal lipoproteins are also depicted as HDL, LDL and VLDL. Corresponding marker apolipoproteins are indicated (apoAI, green; apoB, blue; apoCI, magenta; apoE, orange). NPC1L1 is present at the apical membrane of hepatocytes (red). Since the exact way HCV interacts with this receptor is currently unclear, no virion has been depicted in its vicinity. Photographic credit for TEM images: Perrault M and Pécheur EI, unpublished data.

In terms of receptor recognition, ApoAI is the main apolipoprotein involved in HDL recognition by the scavenger receptor SR-BI [25] (Figure 2). Concerning LDL and VLDL, apoB100 drives their specific recognition by the LDL receptor (LDL-R). Lastly the exchangeable apoE is involved in the recognition of remnant lipoprotein captors such as the VLDL receptor and heparan sulfate proteoglycans [26]. ApoE is also capable of recognition of SR-BI and can therefore participate in HDL binding to this receptor molecule [27].

#### 1.1.2. Arrangement of HCV Virions

The viral nucleocapsid, formed by the core protein and RNA, was found in fractions enriched in apolipoprotein B (apoB), triglycerides, and cholesterol esters, hallmarks of beta-lipoproteins of low to very low density (LDL and VLDL, resp.). The concept of lipo-viro-particle (LVP) was therefore put forward [19, 20]. This gained further support with the characterization of a physical association of apoB and apolipoprotein E (apoE) with HCV RNA-positive particles, and with the isolation of LVPs containing the viral glycoproteins E1 and E2 [28]. A most recent thorough characterization of low density fractions from serum of HCV-infected patients firmly established the physical association within LVP of apolipoproteins B, CI, CII, and CIII and E, HCV RNA, and E1 and E2 glycoproteins [29]. The presence of these apolipoproteins, in the absence of apoAI and AII, is a hallmark of LDL and VLDL [3, 22]. This study further revealed that LVPs and triglyceride-rich lipoproteins (TRL) LDL and VLDL have a similar lipid composition, in terms of both phospholipid and triglyceride species. It was finally observed that LVPs in the blood of patients circulate as a mixed population of particles, most of them being subviral particles devoid of viral nucleocapsid but positive for HCV E1/E2 glycoproteins [29]. Such incomplete or empty LVPs (eLVPs) had previously been isolated and characterized from a cell culture model system and displayed a typical aspect of TRL [30, 31]. In the current absence of high-resolution structure of any of these particles, a schematic representation is presented in Figure 1(c) [see also [3]]. This surprising observation now made from patients' blood samples raises the hypothesis that these subviral eLVPs could help HCV escape the immune system, and might therefore play a major role in chronicity and its long-term evolution. Also this is the first description of such a dual structure for a viral particle, that could (partly) explain HCV adaptation to humans, and play a key role in the physiopathology of the infection [32, 33].

Interestingly, HCV RNA associated with low-density fractions from plasma of infected chimpanzees displayed the highest infectivity, compared to higher density fractions that were only poorly infectious [34]. Similar observations were achieved with HCV viral particles produced in cell culture (HCVcc) [35], where low-density fractions displayed the highest specific infectivity (defined as the amount of infectivity to the total number of virus particles or genomes in the sample) and specific fusogenicity (defined as fusion activity per given quantity of HCV core protein) [36].

Analyses of lipid compositions of low density virions from infected patients and from cultured cells revealed common features, and similarities to serum LDL and VLDL [29, 37]. Dissimilarities were conversely obvious from the analyses of apolipoprotein content: serum LVPs contained apoB and apoE, whereas apoB was not found as an integral component of HCVcc ([38–40]; reviewed in [41]). Indeed HCVcc capture with apoB-specific antibodies was inefficient, suggesting no or sporadic association of these virions with apoB, while anti-apoE antibodies efficiently retained viral particles [3, 37]. Therefore, although HCVcc are a model of choice to study HCV life cycle, their dissimilarities to serum HCV must be kept in mind when studying HCV reactivity toward lipid receptors and interplay with lipid metabolism.

### 1.2. HCV Cell Surface Receptors Involved in Lipid Metabolism

For a thorough description of all known HCV receptors and their involvement in HCV cell entry, we refer the reader to recent reviews and references therein [8, 41–47].

Here we will focus on molecules described as HCV receptors or entry factors that are physiologically involved in lipid metabolism: the receptor for low-density lipoproteins (LDL-R), the receptor for high-density lipoproteins scavenger receptor BI (SR-BI), and the recently described cholesterol-uptake receptor Niemann-Pick C1-like 1 (NPC1L1). Before that, these receptors have to be replaced in their cellular context: the hepatocyte. 

#### 1.2.1. Architecture of Hepatocytes

 Hepatocytes are the main functional cells of the liver and perform several metabolic, endocrine, and secretory functions [48]. They contribute to roughly 85% of the liver mass, and they are epithelial cells with a unique architecture as compared with other epithelial cells. The cells are polygonal in shape and their sides can be in contact either with blood sinusoids (through their sinusoidal face) or neighbouring hepatocytes (through lateral faces) (Figure 2). A portion of the lateral faces of hepatocytes is modified to form bile canaliculi [49]. Microvilli are present abundantly on the sinusoidal face and project into the lumen of the bile canaliculus. This biliary pole forms the apical membrane delimited by adherens junctions, tight junctions, and desmosomes (Figure 2). Conversely, the blood pole does not sit on a basal lamina, in contrast with simple epithelia, but it is in contact with the hepatic extracellular matrix. 

At the basolateral/blood pole, hepatocytes capture nutrients, substances to be transported to the bile, and several receptors or effectors such as integrins, transporters of nutrients, and tetraspanins are concentrated at this membrane. Lipoprotein receptors such as the LDL-R and SR-BI are present at this pole. At the apical pole, hepatocytes secrete bile components and reabsorb other molecules, like free cholesterol. Membrane proteins such as transporters of bile acids and multidrug-resistance proteins (ATPase pumps for various substrates) and the cholesterol absorption factor NPC1L1 are localized at this pole [50, 51]. The apical membrane surface is small compared to that of the basolateral membrane. Tight junctions are formed concomitantly to the apical pole, and the development of membrane polarity is integral to the process of hepatocyte differentiation through cell-cell contact, cell-extracellular matrix contact, or both [52, 53]. Therefore the formation of these specialized membrane domains implies different protein compositions at the apical and basolateral poles.

In this context, the relative position of LDL-R, SR-BI, and NPC1L1 is schematized in Figure 2.

#### 1.2.2. The Low-Density Lipoprotein Receptor (LDL-R)

Pioneering studies reporting the involvement of the LDL receptor (LDL-R) in the cellular entry of HCV came out in 1999 [54–56]. This receptor transports cholesterol-rich LDL from the extracellular medium into cells, *via* clathrin-mediated endocytosis, thereby increasing the levels of intracellular cholesterol. Accordingly LDLR knockout mice display a marked elevation in apoB and in total plasma cholesterol levels compared to wild-type animals [57].

Competition experiments between serum HCV and human lipoproteins showed that LDL and VLDL, but not HDL, efficiently inhibited HCV binding and subsequent endocytosis by cells, in a dose-dependent manner. Interestingly, pretreatment of VLDL by antibodies to apoB and apoE, although inhibiting VLDL uptake, did not fully block concomitant HCV entry in these competition experiments, suggesting a direct uptake of viral particles by the LDL-R [54]. However, these results were obtained on cell lines unable to sustain HCV infection. The LDL-R was also found to be required for HCV adsorption to the cell surface [58].

The direct implication of LDL-R in HCV entry was later revealed by the Maurel's group, using a more physiological model for HCV infection, that is, primary human hepatocytes (PHH) infected by serum HCV [59]. LDLs were confirmed to negatively compete with HCV entry and infection; furthermore, competition experiments using either recombinant soluble peptides corresponding to the LDL-R ectodomain or monoclonal antibodies against similar domain revealed a dose-dependent inhibition of HCV entry into PHH. Also silencing the LDL-R mRNA ablated ligand uptake and reduced HCV infection of hepatoma cells, whereas infection was rescued upon cell ectopic LDL-R expression [60]. This gave strong support to the concept of LDL-R playing a key role in HCV entry and infection (Figure 2).

Recent data obtained with HCVcc and human hepatoma cells (Huh-7) confirmed that LDL-R depletion reduced HCV infectivity, and that a soluble form of LDL-R competed with HCV binding to this receptor and its subsequent entry into hepatocytes [61]. Virion pretreatment with lipoprotein lipase, a triglyceride hydrolase, and a bridging factor for LDL-R-mediated lipoprotein uptake reduced overall HCV infectivity but increased RNA internalization, suggesting that LDL-R-mediated HCV internalization led to a nonproductive pathway of infection. Also kinetics of LDL-R-dependent lipoprotein internalization were much faster than those of HCV. These data would therefore tend to demonstrate that LDL-R is not an essential factor for HCV entry. However, one must keep in mind that HCVcc can be considered “incomplete” LVPs since they lack apoB, and human hepatoma cells such as Huh-7 produce apoB-positive lipoproteins predominantly in the LDL range, indicating a defect in VLDL secretion [40, 62]. These data might therefore not be fully relevant from a physiopathological point of view.

Proprotein convertase subtilisin/kexin type 9 (PCSK9) is a key enzyme involved in cholesterol homeostasis. It is known to enhance the degradation of hepatocyte LDL-R and VLDL-R [63, 64]. In the light of LDL-R involvement in HCV entry and infection, the potential role played by PCSK9 in HCV infection has been scrutinized. *In vitro*, LDL-R expression was downregulated in hepatoma cells transiently expressing PCSK9, and, *in vivo*, mice knocked out for *Pcsk9* displayed a drastic reduction in their liver expression level of LDL-R. Concomitant to LDL-R expression downregulation, cell-cultured HCV infection was impeded in a dose-dependent manner by addition of soluble PCSK9 to hepatoma cells, while cells stably expressing PCSK9 at their surface became resistant to HCV infection [65]. Also HCV E1-dependent cell entry of pseudotyped particles was impaired in hepatoma cells transiently expressing PCSK9 [66]. These data further reinforce the view that HCV entry and subsequent infection relies on hepatocyte surface expression of LDL-R. 

Studying the expression profiles of genes involved in lipid metabolism and HCV entry in liver biopsies from HCV-infected patients with chronic hepatitis C, the group of Enjoji revealed that LDL-R gene expression was significantly suppressed in HCV-infected compared with normal liver, and inversely correlated to the serum content of LDL-associated cholesterol and HCV core protein [67, 68]. Interestingly, a positive correlation between LDL-R and SR-BI gene expression was observed only in HCV-infected livers. Taken together, these data point to a strong interplay between HCV infection and modulation of lipoprotein receptor expression.

#### 1.2.3. The Scavenger Receptor BI (SR-BI)

 This molecule is known for long as an authentic receptor for high density lipoproteins (HDLs). It also plays a role in VLDL metabolism and could act as a receptor for these lipoproteins as well [69]. Its role in HCV entry was suggested ten years ago, with data showing that a soluble fragment of HCV E2 (sE2) could bind to the surface of hepatoma cells. This led to the isolation of SR-BI [70]. Since then, SR-BI has considered a necessary and sufficient receptor for HCV entry, together with CD81, claudin-1, and occludin [71]. 

Binding of sE2 to SR-BI could be inhibited by an anti-SR-BI serum [72]. Conversely, expressing SR-BI at the surface of cells not permissive for HCV infection led to specific binding of HCV sE2 [70, 73]. A cell-cultured virus bearing a mutation in the E2 glycoprotein (G451R) exhibited an altered dependence on SR-BI for hepatocyte infection [73], suggesting that SR-BI recognition by HCV depends on E2. Conflicting results were obtained using viral particles from the serum of HCV-infected patients: HCV/SR-BI interaction was indeed reported to rely on the lipoprotein part of virions, namely, apoB [74]. Once again, such discrepancy could come from the use of virions from different origins. However, recent data analyzing the molecular details of HCV/SR-BI association showed that determinants of this interaction were different depending on the density of virion population considered [75]. Lower density HCV subpopulations made usage of SR-BI through its lipid transfer function and in an E2-dependent manner leading to enhancement of cell entry. Intermediate density virions used SR-BI during their initial phase of hepatocyte attachment; this was apo-E-dependent but E2-independent. Finally the SR-BI-mediated entry step *per se* was found independent of HCV density, but dependent of SR-BI lipid transfer function. This tends to reconcile previous data and demonstrate that lipoprotein and viral components of the virion play an active role in SR-BI-mediated entry into hepatocytes (Figure 2). 

The implication of SR-BI in the early steps of HCV infection of hepatoma cells and PHH was further demonstrated using strategies based upon antibodies to SR-BI and gene extinction [72, 76–78]. Recently, human monoclonal antibodies to SR-BI were generated, that prevented infection of hepatoma cells and PHH by HCVcc. Some of these antibodies also efficiently impeded intrahepatic spread of serum-derived HCV, inoculated to immunosuppressed chimeric mice with humanized liver [79, 80]. Altogether these data define SR-BI as an essential factor for HCV entry into hepatocytes.

Cell lines where SR-BI ectopic expression restored HCV infection allowed to demonstrate that the lipid transfer function of SR-BI should be intact to convey HCV entry [81]. Since SR-BI is an HDL receptor, several investigations dealt with the relationship between its capacity to bind HDL and its involvement in HCV entry. HDLs were found to enhance HCV infectivity [76, 82–85] (for a specific review, see [47]), although no direct interaction between HCV and HDL could be observed [82, 85]. Anti-SR-BI antibodies abrogated this enhancing effect [76, 78, 86]. Nevertheless, how HDL enhances HCV infectivity remains unclear. A role for apoCI has been suggested [75, 84, 87, 88]. ApoCI is an exchangeable apolipoprotein mainly associated with VLDL (see above), but a minor component of HDL [89]. It is therefore present at the surface of both plasma HDL and LVP virions, and could thereby play a role in HCV entry, notably through an enhancement of viral membrane fusion properties [84]. Moreover, recent work points that different regions of SR-BI are involved in the recognition of HDL and HCV; indeed certain mutant SR-BI receptor proteins were defective in binding the soluble form of HCV E2, while displaying a wild-type phenotype for HDL recognition and mediating an increased cholesterol efflux as compared to native SR-BI [90]. At that stage one could only speculate that HDL could enhance HCV infectivity through a concomitant binding of HDL and HCV on sites of SR-BI in close vicinity but distinct, in an apoCI-dependent manner (Figure 2). 

PDZK1 is an adaptor protein linking the cytosolic domain of transmembrane proteins to other cellular partners essential for their normal functioning. It binds to SR-BI, regulates its levels in the liver, and controls HDL metabolism [91]. Its indirect role as a facilitator of SR-BI-mediated HCV entry was recently demonstrated [92]. Hepatoma cells stably knocked down for PDZK1 expression became less susceptible to HCV infection; this effect on HCV infectivity was related to PDZK1 ability to bind SR-BI. 

Altogether these data establish SR-BI as an essential and multifaceted entry factor for HCV, playing a role through direct interactions with the lipoprotein and viral components of the particles, through its lipid transfer function and through intracellular partners with which it interacts. They also pinpoint a subtle relationship between virion structure and receptor recognition.

#### 1.2.4. The Niemann-Pick C1-Like 1 Cholesterol Absorption Receptor (NPC1L1)

 All the above data point to a tight entanglement between lipoprotein/cholesterol metabolism, HCV structure, and its early steps of infection. Further evidence of such a link was provided most recently by the group of Uprichard ([93], and recent specific reviews in [45, 94]). 

The Niemann-Pick C1-like 1 cholesterol absorption receptor (NPC1L1) is an integral protein located at the apical pole of intestinal enterocytes and of hepatocytes, at the bile canaliculus membrane. In the liver, it transports free excreted cholesterol from the bile to intracellular compartments. It is therefore an essential player in cholesterol homeostasis. Its involvement as an HCV entry factor was evidenced from the following observations: (i) its depletion by RNA silencing or its blocking by a specific antibody led to a drastic reduction in HCV infectivity; (ii) it was downregulated with time in HCV-infected cells; (iii) an inhibitor of its internalization, ezetimibe, impeded HCV entry *in vitro* in a dose-dependent manner, and delayed initial HCV infection *in vivo *in mice with humanized liver (see above); (iv) lastly, its implication in HCV entry was correlated to the amount of virion-associated cholesterol, implying again that the virion structure as an LVP is a key to the binding/recognition step [93] (Figure 2). 

This last point is therefore common to the recognition by HCV of all its receptors described to date that are involved in cholesterol/lipoprotein metabolism. The “subversion” of cholesterol receptors by HCV to its own profit is likely to play a major role in the physiopathology of the infection on the long term [33]. Indeed chronic hepatitis C is often accompanied by symptoms of lipid metabolism disorders such as low levels of plasma total cholesterol and liver steatosis.

### 1.3. Enzymes Involved in Lipid Metabolism and HCV Entry

Tight links therefore exist between HCV entry and infection, and receptor molecules involved in lipid metabolism. The implication of lipid-synthesizing and lipid-modifying enzymes will be examined in the following.

#### 1.3.1. Fatty Acid Synthase (FASN)

 This enzyme (EC 2.3.1.85) is a whole enzymatic system that catalyzes fatty acid synthesis, therefore *de novo* lipogenesis. It is thus a key player in lipid metabolism. 

*In vitro* analyses revealed that HCV core protein induced *FASN * promoter activation [95]. The implication of FASN at early stages of HCV infection was then evaluated by the group of Yang [96]. FASN was found to co-fractionate with HCV virions, together with apoE and HCV core, although a direct interaction between FASN and viral particles could not be established. Pharmacological or gene inhibition of FASN blocked the entry of HCV pseudotyped particles into hepatoma cells and down-regulated the expression of claudin-1, an essential receptor of HCV entry. Furthermore, FASN expression was upregulated in HCVcc-infected cells compared to normal cells. This identified FASN as a regulator of HCV entry into hepatocytes and as an important player on the pathway leading to infection.

Interestingly, suppression of the *FASN* gene in hepatocarcinoma cell lines reduced their proliferation, suggesting a role of aberrant lipidogenesis in the pathogenesis of hepatocarcinoma [97]. In the liver of chronically HCV-infected patients, the transcriptional level of expression of the *FASN* gene was found to be upregulated [67]. In addition, serum levels of FASN were significantly increased in patients with chronic hepatitis C and correlated with the degree of liver steatosis [98].

Altogether, these data establish FASN as an essential player at several stages of HCV infection, in particular at the earlier steps of entry. As noted above for other players, subversion of this key enzyme of lipid metabolism at early stages of infection could be later translated into the lipid disorders associated to chronic hepatitis C and hepatocarcinogenesis.

#### 1.3.2. Lipoprotein Lipase (LPL)

 Lipoprotein lipase (EC 3.1.1.34) is a soluble enzyme, existing as a circulating form in the plasma, and as a membrane-attached form at the surface of several cell types (endothelial cells, hepatocytes, etc.). The plasma LPL pool is very little, compared to what is retained at cell surfaces by heparan sulfate proteoglycans. LPL has a dual function: (i) it hydrolyzes triglycerides in lipoproteins and thereby converts VLDL and chylomicrons into lipoproteins of intermediate to low density (catalytic function); (ii) it mediates hepatic uptake of VLDL and chylomicrons by making a link between the lipoprotein on one side and heparan sulfate proteoglycans on the other side (bridging activity). ApoCII is a cofactor of its activity.

Pioneering work from Thomssen showed that LPL disrupted the structure of serum HCV particles, but not of HBV [99]. This led to HCV RNA degradation, suggesting the degradation of virion-associated lipid components. These experiments were performed with LPL from *Pseudomonas aeruginosa*, not likely to behave as human LPL. Nevertheless, corroborating observations were done using bovine LPL; treatment of cell-cultured HCV particles by the enzyme shifted the particles to higher densities, lowered the amount of virion-associated apoE, and reduced HCV infectivity [100]. This puts forward the catalytic function of LPL on HCV. 

The influence of LPL on HCV infection *in vitro* was further evaluated by the group of Budkowska, which showed that LPL-treated hepatoma cells were less permissive to serum HCV infection, in spite of a higher yield of particle binding and internalization. The hypothesis was therefore made of an LPL-stimulated nonproductive route of HCV entry [101]. Later studies, in particular by electron microscopy, revealed that LPL blocked HCV entry by immobilizing viral particles at the cell surface. This mainly relied on the bridging function of LPL [102]. LPL could thus be considered as an anti-HCV factor *in vitro*. 

Could this be transposed to any *in vivo* situation? In a study performed on clinical samples, the group of Young showed that LPL lipolytic activity was inversely correlated to plasma HCV viral loads [103], revealing that LPL is also an anti-HCV factor *in vivo*. In addition, normal VLDL (from healthy donors), plus LDL and VLDL fractions of lipo-viro-particles extracted from the serum of HCV-infected patients, inhibited the lipolytic activity of LPL in *in vitro* analyses. In contrast, normal LDL did not exhibit any inhibitory effect. This suggested that a component of normal VLDL, LDL-LVPs, and VLDL-LVPs could block this activity. This component was identified as apoCIII, absent from the surface of normal LDL, but present at the surface of normal VLDL, and of LDL-LVPs and VLDL-LVP fractions in HCV-infected patients. From these complex data, the following picture therefore emerges. ApoCIII has an intrinsic anti-LPL activity; under physiological conditions, VLDLs are transformed into LDL by LPL, with a balance in favor of LDLs that do not inhibit LPL activity. In HCV-infected patients, VLDL-LVPs would be transformed into LDL-LVPs, positive for apoCIII and therefore different from normal LDL in terms of physico-chemical properties. These LDL-LVPs, together with VLDL-LVPs, could inhibit the LPL catalytic function, through their apoCIII moiety. This would facilitate the propagation and persistence of HCV infection. 

These data should be connected to the observations by Scholtes et al., that LVPs purified from patients serum bear a heavier protein load than their normal lipoprotein counterpart [29]. This additional load could thus be apoCIII. This study also demonstrated the presence in HCV-positive sera of high amounts of noninfectious nucleocapsid-free LVPs (empty LVPs) that might also bear apoCIII. They could play a major role at inhibiting LPL, while infectious (complete) LVPs could invade cells. 

Consequently, any therapy activating the function of LPL would then constitute a promising way to combat HCV infection.

## 2. Conclusion

The intimate adaptation of the hepatitis C virus to its human host is quite unique in the viral world. This could be related to the tight links that the virus weaves with the lipid metabolism of its host, and specifically of its host cells, the hepatocytes. SR-BI, a key receptor of lipoproteins, is an essential factor of HCV entry. The depletion of several major actors of lipoprotein or cholesterol metabolism, such as SR-BI, LDL-R, and NPC1L1, has led to the conclusion that they are involved in HCV entry and early steps of infection. From that, one could legitimately ask the questions of “why and how.” Why does this virus make usage of so many molecules, that, although not redundant, are all playing a role in very close metabolic pathways? Why does the depletion of anyone of these factors lead to an almost complete loss of HCV infectivity, even when other receptor molecules remain unaffected? How do these receptors and enzymes function together for viral entry? How could the hepatotropism of HCV be explained, since none of the molecules described here are specific to hepatocytes or to a liver-specific function? These questions and others therefore remain open on promising perspectives of answers in terms of therapeutic options to combat and, possibly, eradicate HCV infection.
